# The association between diabetic retinopathy and cognitive impairment in patients with diabetes mellitus: a systematic review and meta-analysis

**DOI:** 10.3389/fendo.2026.1881602

**Published:** 2026-10-15

**Authors:** Omar Subih, Wejdan Abdullah Al Mustafa, Tareq Ahmed AlOmem, Abdulaziz Mohammed Alrasheed, Maha Saeed Altunaiji, Aryam Abdullah Mubarak Alhasan, Abdulaziz Ibrahim Atef Alshehri, Bader Muzhier Alshahrani, Ruwaym Jaber Mohammed Alharbi, Ahmad Moahmmad Alshatti, Manar Eid Al Mutairi, Samar Mussari Alkhaldi, Inam N. Bifari

**Affiliations:** 1 College of Medicine, Alfaisal University, Riyadh, Saudi Arabia; 2 Ophthalmology Department, Imam Abdulrahman Bin Faisal University, King Fahad Hospital of The University, Dammam, Saudi Arabia; 3 College of Medicine, King Saud University, Riyadh, Saudi Arabia; 4 Taif Armed Forces Hospital, Taif, Saudi Arabia; 5 College of Medicine, University of Sharjah, Sharjah, United Arab Emirates; 6 College of Medicine, Jouf University, Al-Jouf, Saudi Arabia; 7 Al-Namas General Hospital, Al-Namas, Saudi Arabia; 8 Department of Neurology, Security Forces Hospital, Riyadh, Saudi Arabia; 9 Kuwait Institute for Medical Specialization, Kuwait City, Kuwait; 10 Faculty of Medicine, Hail University, Hail, Saudi Arabia; 11 College of Medicine, Taif University, Taif, Saudi Arabia; 12 Alhada Armed Forces Hospital, Taif, Saudi Arabia

**Keywords:** Alzheimer’s disease, cognitive impairment, dementia, diabetes mellitus, diabetic retinopathy

## Abstract

**Background:**

Diabetic retinopathy (DR) is a recognized microvascular complication of diabetes in which neurovascular and neurodegenerative mechanisms may also contribute. This systematic review evaluated the association between DR and cognitive impairment or dementia, including Alzheimer’s disease and vascular dementia, using evidence from cross-sectional and longitudinal observational studies.

**Methods:**

We searched for relevant articles up to December 2025 from PubMed, Scopus, Web of Science, and Cochrane Library. Two independent reviewers extracted data from the selected studies, including baseline information and outcomes. All quantitative syntheses were conducted using R version 4.4.1.

**Results:**

Fourteen studies with reported study populations totaling 2,871,847 participants were included. Seven studies contributed to the crude dichotomous synthesis, which did not demonstrate a statistically significant association between diabetic retinopathy and cognitive impairment or dementia (OR 1.07, 95% CI 0.94–1.23; p=0.310; I^2^=52.3%). Seven studies contributed adjusted longitudinal estimates for incident dementia; diabetic retinopathy was associated with a higher incidence of dementia (HR 1.55, 95% CI 1.19–2.02; p=0.001; I^2^=97.8%; 95% prediction interval 0.65–3.68). Evidence concerning Alzheimer’s disease and vascular dementia remained inconsistent.

**Conclusion:**

Longitudinal evidence suggests that diabetic retinopathy is associated with a higher incidence of dementia, although substantial heterogeneity and the observational evidence base limit confidence in the pooled estimate. Evidence concerning Alzheimer’s disease and vascular dementia remains inconclusive.

**Systematic review registration:**

https://doi.org/10.17605/OSF.IO/YS462, identifier YS462.

## 1 Introduction

Retinopathy, neuropathy, and nephropathy are classically recognized complications of diabetes mellitus (DM) (1). However, there has been a growing body of literature addressing the associated cognitive decline, mild cognitive impairment (MCI), and dementia in patients with diabetes (2), with multiple studies reporting an association between DM and dementia subtypes, Alzheimer’s disease (AD), and vascular dementia (VaD) (3–5). Contemporary syntheses remain equivocal in determining the exact underlying mechanism through which diabetes impacts cognitive functions, with various theories implicating microvascular complications, glycemic strain, systemic cardiometabolic comorbidities, neuroinflammation, or other neurodegenerative processes (3, 6, 7).

While previously considered a microvascular complication of diabetes, evidence suggests that DR is attributable to both microvascular insults and neurodegeneration, such as inner retinal neurodegeneration, manifested through ganglion cell depletion, reactive gliosis, inner retinal thinning, and functional deficits observed via electroretinogram (8). In addition, these neuro-retinal impairments often manifest prior to the emergence of clinically detectable microvascular lesions (9). These findings necessitate a paradigm shift in our understanding of DR, suggesting that the condition is a neurodegenerative ocular disease that initiates during the earliest phases of diabetic pathology (10, 11). Early impairment of the neurovascular unit in the retina of patients with diabetes is evidenced by diminished vasomotor responses to hyperoxic and flickering light stimuli, reflecting a systemic regulatory breakdown rather than isolated vascular or glial damage. These preclinical disturbances are analogous to neurovascular dysfunction seen in cerebral pathologies like cerebrovascular insults and AD (8, 12, 13).

Research indicates that type 2 DM significantly alters both the physical structure and functional activity of the brain (14). Structurally, MRI scans reveal that diabetic individuals often experience brain atrophy, particularly in the hippocampus and temporal lobe, alongside a higher prevalence of small infarcts and hyperintense lesions in the white matter (15). Even in the early stages of cognitive decline, functional imaging shows that diabetes disrupts neural signaling in regions responsible for emotion and thought, such as the frontal lobe and amygdala, while also causing abnormal activity in the visual processing centers (16, 17).

Prior meta-analyses were met with several limitations. For instance, one meta-analysis could not determine whether the association between DR and CI was higher with increasing severity of DR, such as in patients with proliferative DR, due to the mixed nature of the analyzed data. Moreover, inconsistent adjustments for confounding factors across pooled data precluded combining the estimates in a multivariate model (18). In another meta-analysis, the variability in the tools used to assess cognitive function confounded the pooled analysis, with high heterogeneity attributed to multiple factors, including the type of diabetes, disease duration, and other demographic factors (19). Moreover, the underpowered estimates for some subtypes of dementia, such as VaD, prevented firm conclusions. Various sources of bias also existed, including survival bias, diagnostic biases due to self-reported data, and biases due to the reliance on medical records, which skews the results toward treated patients (20, 21). Accordingly, this updated review evaluated the association between DR and cognitive impairment or dementia using a search extending through 2025. Unlike previous reviews, we separated crude dichotomous associations from adjusted longitudinal hazard estimates, verified that population-based studies provided an explicitly DR-specific or diabetes-restricted estimate, incorporated the recently published Lee et al. cohort, and summarized Alzheimer’s disease and vascular dementia findings separately.

## 2 Methods

Following the guidelines outlined in the Cochrane Handbook for Systematic Reviews of Interventions and the Preferred Reporting Items for Systematic Reviews and Meta-Analyses (PRISMA), we conducted a meta-analysis to examine the relationship between DR and CI/dementia in patients with diabetes (22, 23). The review protocol was registered on the Open Science Framework (OSF) and is available at: https://doi.org/10.17605/OSF.IO/YS462.

### 2.1 Eligibility criteria

Eligibility was defined using the PICOS framework, with diabetic retinopathy treated as the exposure. Population: adults with type 1 or type 2 diabetes from diabetes-specific cohorts, or participants in population-based cohorts providing an explicitly diabetic-retinopathy–specific estimate; Exposure: diabetic retinopathy identified through clinical examination, retinal photography, validated grading systems, medical records, or diagnostic codes; Comparator: participants without diabetic retinopathy or the study-defined reference group for the diabetic-retinopathy–specific estimate; Outcomes: cognitive impairment, mild cognitive impairment, all-cause dementia, Alzheimer’s disease, or vascular dementia assessed using validated cognitive tests, established diagnostic criteria, medical records, or diagnostic codes; and Study design: full-text, English-language cross-sectional, prospective cohort, or retrospective cohort studies. Quantitative synthesis required sufficient event data to calculate an odds ratio or a reported hazard ratio with a corresponding measure of uncertainty. We excluded non-human studies, duplicate reports, case reports, case series, conference abstracts, letters, editorials, commentaries, reviews, meta-analyses, and studies without an eligible exposure, comparator, cognitive outcome, or extractable effect estimate. Studies reporting only nonspecific retinopathy without establishing a diabetic etiology or providing a diabetic-retinopathy–specific estimate were also excluded.

### 2.2 Information sources and search strategy

A preliminary scoping search of PubMed, Scopus, and Embase was used only to identify candidate keywords and controlled-vocabulary terms. Embase was not part of the formal systematic retrieval set and was therefore not included in the PRISMA record counts. The formal systematic search was conducted in PubMed, Scopus, Web of Science, and the Cochrane Library from database inception through December 2025. The strategies combined controlled-vocabulary and free-text terms describing diabetes mellitus, diabetic retinopathy or diabetic retinal disease, cognitive impairment, cognitive decline, mild cognitive impairment, dementia, Alzheimer’s disease, and vascular dementia. The complete database-specific strategies, including search dates, fields, Boolean operators, filters, and limits, are provided in **Supplementary Table S2**. Backward and forward citation searching was also performed for eligible reports. Trial registries, theses, dissertations, and preprint servers were not systematically searched.

### 2.3 Selection process

Two reviewers independently screened the titles and abstracts of all unique records against the predefined eligibility criteria. The same reviewers then independently assessed the full texts of potentially eligible reports. Disagreements at either stage were resolved through discussion and consensus; when consensus could not be reached, a third reviewer adjudicated the decision.

### 2.4 Data collection

Two reviewers independently extracted data into a standardized shared spreadsheet using Google Sheets. Extracted items included first author, publication year, country, study design, data source and study period, sample size, participant age and sex, diabetes type, definition and severity of diabetic retinopathy, comparator definition, cognitive outcome and ascertainment method, follow-up duration, effect measure, adjusted covariates, and quantitative data required for synthesis, including event counts, odds ratios, hazard ratios, 95% confidence intervals, and p values. After extraction, the reviewers cross-checked the entries and resolved discrepancies by consensus; unresolved disagreements were adjudicated by a third reviewer. Missing or unclear information was recorded as not reported.

When a study reported multiple eligible estimates, we selected the most fully adjusted model that retained the eligible DR exposure, comparator, and cognitive outcome. DR-specific estimates were preferred over composite diabetic-eye-disease estimates, and diabetes-restricted estimates were preferred when the overall cohort was population based. For Lee et al., the time-dependent DMR estimate from Model 3 was selected. For Shang et al., the DR-specific analysis restricted to participants with diabetes was selected rather than the broader DRED estimate. The study-level numerical data and the source of every estimate used in the quantitative syntheses are presented in **Table 1**. Accordingly, the Hwang and Lee population-based cohorts were retained because they reported explicit DR/DMR estimates; they were not treated as direct DR-versus-no-DR comparisons within exclusively diabetes-only cohorts.

**Table 1:** Study-level numerical data used in the quantitative syntheses of diabetic retinopathy and cognitive outcomes.

Study	Outcome	Exposure	Comparator	DR events/total	Comparator events/total	Effect used (95% CI)	Estimate type/source	Weight
Crosby-Nwaobi 2013 (25)	Positive cognitive screen	PDR	No/mild DR	6/128	31/252	OR 0.35 (0.14–0.86)	Crude OR derived from Table 3	2.2%
Gao 2016 (30)	Dementia	DR	No DR	40/325	92/784	OR 1.06 (0.71–1.57)	Crude OR derived from **Table 2**	11.4%
Kimura 2008 (33)	Dementia	DR	No DR	14/106	32/280	OR 1.18 (0.60–2.31)	Crude OR derived from **Table 2**	4.0%
Murata 2017 (27)	MCI or dementia	Retinopathy	No retinopathy	20/51	58/196	OR 1.54 (0.81–2.91)	Crude OR derived from **Table 1**	4.4%
Xia 2020 (28)	Dementia	DR	No DR	28/146	19/151	OR 1.65 (0.88–3.11)	Crude OR derived from **Table 2**	4.5%
Bruce 2014 (24)	Incident dementia	Baseline DR	No baseline DR	4/30	13/290	OR 3.28 (1.00–10.78)	Crude OR derived from Table 3	1.3%
Hendrie 2018 (31)	Dementia	Retinopathy	No retinopathy	302/1,698	611/3,530	OR 1.03 (0.89–1.20)	Crude OR derived from Table 3	72.2%
Deal 2019 (26)	Incident all-cause dementia	Moderate/severe DR	No retinopathy	—	—	HR 1.86 (1.36–2.55)	Adjusted diabetes-restricted estimate	13.8%
Exalto 2014 (29)	Incident dementia	Severe diabetic retinal disease	Absent	—	—	HR 1.37 (1.22–1.53)	Adjusted model, Table 4	16.5%
Lee 2025 (37)	Incident dementia	Time-dependent DMR	Study-defined no-DMR reference group	—	—	HR 2.74 (2.44–3.08)	Model 3; original value 2.738 (2.437–3.075)	16.5%
Rodill 2018 (34)	Incident dementia	Time-updated DR	No DR	—	—	HR 1.12 (0.82–1.53)	Adjusted Model 3	13.8%
Yu 2020 (36)	Incident all-cause dementia	Coded DR	No coded DR	—	—	HR 1.09 (1.07–1.11)	Fully adjusted model	17.0%
Hwang 2021 (32)	Incident all-cause dementia	Recorded DR	Study-defined no-recorded-DR reference group	—	—	HR 1.51 (0.70–3.27)	Adjusted population-based estimate	7.0%
Shang 2023 (35)	Incident dementia	DR among participants with diabetes	No DR among participants with diabetes	—	—	HR 1.61 (1.30–2.00)	DR-specific diabetes analysis	15.3%

### 2.5 Outcome measures

#### 2.5.1 Cognitive impairment

Cognitive outcomes included objectively assessed cognitive impairment, mild cognitive impairment (MCI), all-cause dementia, Alzheimer’s disease, and vascular dementia. MCI and dementia were not considered clinically equivalent. The crude dichotomous synthesis used one eligible study-defined cognitive endpoint from each report and was therefore interpreted as a broad exploratory synthesis of cognitive impairment or dementia. The longitudinal synthesis used adjusted estimates for incident dementia. Alzheimer’s disease and vascular dementia were summarized separately because insufficient comparable data were available for subtype-specific meta-analysis. Eligible outcomes were ascertained using validated cognitive instruments, established clinical criteria, medical records, or diagnostic codes. Some studies defined CI based on a clinical score cutoff, such as the clinical dementia rating (CDR), with scores of 0.5 indicating CI without dementia, and scores of 1 or more indicating dementia. Other assessment tools, such as the Mini-Mental State Examination (MMSE) score, the Addenbrooke’s Cognitive Examination-Revised (ACE-R), or the Montreal Cognitive Assessment (MoCA) score, were used as well (24–28). Other studies used ICD diagnosis codes to define CI/dementia, the DSM-IV criteria for dementia, or the NINCDS-ADRDA criteria for Alzheimer’s disease, or the NINDS-AIREN criteria for VaD (29–36).

#### 2.5.2 Diabetic retinopathy

DR was defined by grades detected on ophthalmoscopy or retinal photography (24, 30, 33), with the ETDRS criteria (25), modified Airlie House classification (26), or by specific diagnostic codes provided by the ICD-9 (29, 31, 32, 34, 36, 37).

### 2.6 Risk of bias

Because all eligible studies were observational and no randomized controlled trials were included, the Critical Appraisal Skills Programme Randomized Controlled Trial Checklist was not applicable. Cohort and cross-sectional studies were assessed using the NIH Quality Assessment Tool for Observational Cohort and Cross-Sectional Studies (38). To ensure uniform application, two independent reviewers first used the tools on a random sample of studies and refined the assessment criteria. Following this calibration, the same two reviewers independently assessed all studies. Any disagreements were resolved through arbitration by a third reviewer.

### 2.7 Data synthesis and statistical analysis

Crude event-count data and adjusted time-to-event estimates were synthesized separately because they represent different estimands and should not be interpreted as equivalent. For the crude dichotomous synthesis, study-level 2 × 2 data were converted to log odds ratios and corresponding sampling variances using the escalc function in the metafor package. Adjusted hazard ratios and their confidence limits were log-transformed, and standard errors were derived as [ln(upper confidence limit) − ln(lower confidence limit)]/(2 × 1.96). Both syntheses were fitted using inverse-variance random-effects models with restricted maximum-likelihood estimation of τ^2^ and were subsequently back-transformed for presentation. The Hartung–Knapp adjustment was not applied. Heterogeneity was described using I^2^, τ^2^, the χ^2^ test, and 95% prediction intervals; because the χ^2^ test has limited power with few studies, interpretation emphasized I^2^, τ^2^, prediction intervals, and clinical and methodological differences. Statistical significance was defined as a two-sided p<0.05.

Adjusted cross-sectional estimates were not sufficiently and consistently reported on comparable scales; therefore, the dichotomous synthesis was limited to crude event data and interpreted as exploratory. ORs and HRs were not combined in a single meta-analysis. A leave-one-out sensitivity analysis was performed for the longitudinal synthesis by refitting the restricted maximum-likelihood model after omitting each study in turn. Because only seven studies contributed to each synthesis and potential subgroups frequently contained one or two studies, *post hoc* subgroup analysis and meta-regression were not considered sufficiently stable to support reliable inference. Funnel-plot asymmetry was not assessed because each synthesis contained fewer than 10 studies.

## 3 Results

### 3.1 Study selection

The database searches identified 288 records. After removal of 21 duplicates, 267 unique records underwent title and abstract screening, of which 214 were excluded. Fifty-three full-text reports were assessed for eligibility. Thirty-nine reports were excluded: 17 had no eligible cognitive outcome, 12 had no eligible DR exposure, two were conference abstracts, seven were reviews, and one reported nonspecific retinopathy without an eligible DR-specific estimate. Fourteen studies were included in the systematic review and quantitative syntheses (24–37). The flow diagram for study selection is shown in **Figure 1**.

**Figure 1:**
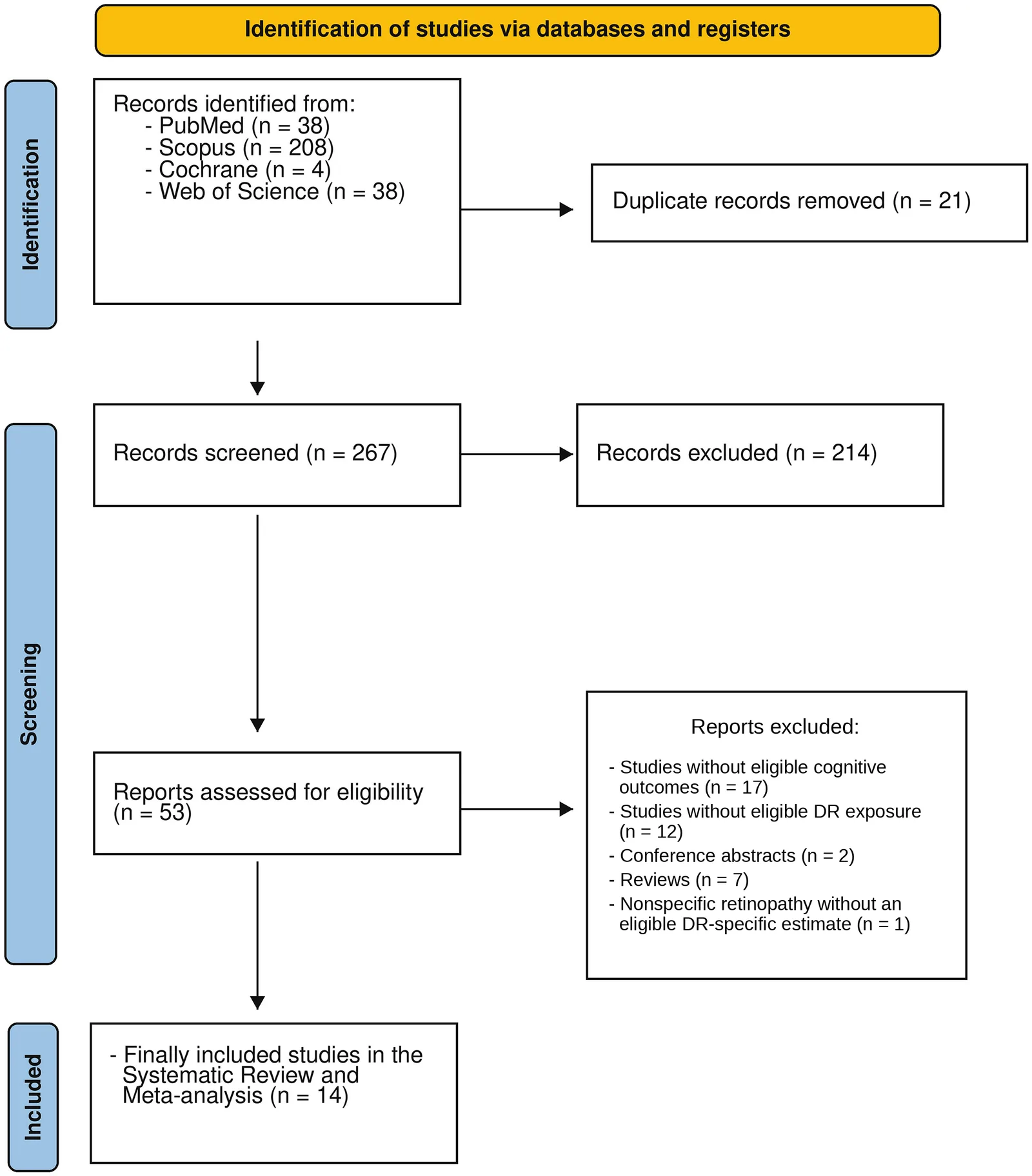
PRISMA flow chart for study selection. This flowchart illustrates the systematic screening and selection process. The initial database search yielded 288 records. The removal of duplicates left 267 unique entries for screening. Title and abstract screening resulted in the exclusion of 214 records. The team evaluated 53 full-text articles for eligibility. After excluding 39 records during the full-text review, 14 studies met all inclusion criteria.

### 3.2 Characteristics of the included studies

The 14 included studies were published between 2008 and 2025 and comprised five cross-sectional and nine longitudinal observational studies, with reported study populations totaling 2,871,847 participants. Follow-up in the longitudinal studies ranged from 4.1 to 15.6 years. Study-level age, sex, population, exposure, and follow-up characteristics are presented in **Table 2**. Hwang et al. and Lee et al. were population-based cohorts with study-defined DR/DMR estimates rather than direct comparisons within exclusively diabetic cohorts.

**Table 2:** Baseline characteristics of the included studies.

Study	Study design	Sample size	Country	Data source	Study period	Age	Female, n (%)	Follow up	Population and eligible analysis	Exposure	Adjustments	Conclusion
Bruce 2014 (24)	Longitudinal observational study	335	Australia	Fremantle Diabetes Study (FDS)	Phase I: 1993–1996; Phase II (Follow-up): 2008–2011	57.5 (9.2)	167 (49.3)	14.7 y	Diabetes-only cohort: adults with type 2 diabetes	DR	Age, sex, education, marital status, BMI, stroke/TIA history, diabetes duration, fasting glucose, insulin therapy, and smoking.	Insulin therapy, diabetic retinopathy, and smoking in mid-life are independent predictors of cognitive impairment and dementia in patients with Type 2 diabetes.
Crosby-Nwaobi 2013 (25)	Cross-sectional study	380	United Kingdom	South East London Diabetic Retinopathy Study (SEL-DRS)/Diabetes Eye Complication Service (DECS)	NA	64.8 (10.8)	166 (43.8)	NA	Diabetes-only cohort: adults with type 2 diabetes; PDR compared with no/mild DR	DR	Age, sex, ethnicity, education, BCVA, diabetes duration, SES, nephropathy, BMI, blood pressure, HbA1c, lipids, alcohol, depression, and emotional distress.	There is an inverse relationship between retinopathy severity and cognitive impairment; patients with proliferative retinopathy (PDR) had better cognitive performance than those with no/mild retinopathy.
Deal 2019 (26)	Population-based prospective cohort study	12,482	United States	Atherosclerosis Risk in Communities (ARIC) Study	Baseline (Visit 3): 1993–1995; Follow-up through 2011–2013	50–73 y	6,929 (56)	15.6 y	Population-based cohort; diabetes-restricted moderate/severe retinopathy estimate used	DR	Age, sex, race, education, BMI, smoking, alcohol, hypertension, diabetes, coronary heart disease, stroke, APOE genotype.	Moderate/severe retinopathy and generalized arteriolar narrowing in mid-life are associated with an increased risk of incident dementia. Retinopathy was associated with cerebrovascular-related dementia etiology but not Alzheimer’s-related etiology.
Exalto 2014 (29)	Longitudinal cohort study	29,961	United States	Kaiser Permanente Northern California (KPNC) Diabetes Registry	Baseline: 1996–1998; Follow-up: 1998–2008	70.6 (6.8)	13,787 (46)	10 y	Diabetes-only cohort: adults aged ≥60 years with type 2 diabetes	DR	Age, gender, race/ethnicity, education, medical utilization, diabetes duration, HbA1c, pharmacotherapy, hyperglycemia/hypoglycemia events, BMI, smoking, and vascular comorbidities (hypertension, lipids, CVD, etc.).	Severe diabetic retinal disease is associated with an increased risk of incident dementia in patients with Type 2 diabetes, suggesting a link between microvascular disease and dementia.
Gao 2016 (30)	Cross-sectional study	1,109	China	Community-dwelling residents in Tianjin	March 2010 – April 2011	72.4 (3.5) y	NA	NA	Diabetes-only cohort: Han Chinese adults aged ≥65 years with type 2 diabetes	DR	Age, gender, education, income, smoking, alcohol, hypertension, hyperlipidemia, CVD history, BMI, HbA1c, diabetes duration, visual impairment.	Conclusion: While Type 2 Diabetes was associated with a high prevalence of Mild Cognitive Impairment (MCI), retinopathy itself was not found to be a statistically significant factor distinguishing between normal cognition, MCI, and dementia groups in the multivariate analysis
Hendrie 2018 (31)	Longitudinal cohort study	5228	United States	Eskenazi Health Services electronic medical records	January 1, 1992 – December 31, 2014	76.6 (4.9) y	NA	13.8 y	Diabetes-only cohort: 5,228 adults with diabetes; Table 3 provided extractable retinopathy–dementia data	DR	Not applicable to the pooled crude OR.	Among 5,228 participants with diabetes, Table 3 reported dementia in 302 of 1,698 participants with retinopathy and 611 of 3,530 without retinopathy. These unadjusted counts were used in the crude dichotomous synthesis.
Kimura 2008 (33)	Cross-sectional study	386	Japan	Kariya Toyota General Hospital	September – November 2003	66.1 (8.2)	160 (41.5)	NA	Diabetes-only cohort: adults aged >50 years with diabetes	DR	Adjusted for age, sex, BMI, diabetes duration	The prevalence of retinopathy was 30.4% in demented subjects vs 27.1% in non-demented subjects; this difference was not statistically significant
Lee 2025 (37)	Longitudinal retrospective cohort study	784,205	South Korea	Korean National Health Insurance Screening Cohort (NHIS-HEALS)	2002–2017	more than 40 years	423,836 (54)	10.9 ± 2.7 y	Population-based cohort with explicitly coded DMR exposure and a study-defined reference group; not an exclusively diabetes-only DR-versus-no-DR comparison	DR	Age, sex, income, visual acuity, systemic comorbidities (HTN, dyslipidemia, stroke, depression, etc.), behavioral factors (smoking, alcohol, BMI), diabetes severity	Patients with DMR had a significantly higher risk of developing dementia (HR 2.74). The risk was higher in those with severe DMR and notably higher in younger patients.
Murata 2017 (27)	Cross-sectional study	281	Japan	Japan Diabetes Clinical Data Management Study (JDDM)	NA	73.4 (6.1) y	137 (48.8)	NA	Diabetes-only cohort: adults aged ≥65 years with type 2 diabetes; retinopathy information available for 247 participants	DR	Multiple regression for MMSE score included NEFA, eGFR, insulin, age, and schooling	Prevalence of retinopathy was not significantly different between dementia (33.3%), MCI (23.9%), and normal cognition (18.4%) groups in this cross-sectional study
Rodill 2018 (34)	Longitudinal cohort study	3742	United States	Kaiser Permanente Northern California (KPNC)	1996–2015	56.1 (8.5)	1,772 (47.4)	6.2 y	Diabetes-only cohort: adults aged ≥50 years with type 1 diabetes	DR	Age, sex, race, HbA1c, acute glycemic events, neuropathy, nephropathy, ESRD, CVD, stroke	In this cohort of older patients with Type 1 diabetes, diabetic retinopathy was not significantly associated with an increased risk of incident dementia (HR 1.12, 95% CI 0.82–1.54)
Xia 2020 (28)	Cross-sectional study	297	China	Tongji Hospital, Tongji Medical College	April 2018 – May 2019	56.8 (6.9)	105 (35.4)	NA	Diabetes-only cohort: adults aged 45–74 years with type 2 diabetes	DR	Age, education, diabetes duration, BMI, HbA1c, lipids, and blood pressure.	Diabetic retinopathy was identified as a significant risk factor for dementia (OR 2.197, P = 0.040) in this cross-sectional analysis, though it was not related to Mild Cognitive Impairment (MCI)
Yu 2020 (36)	Retrospective population-based cohort study	1,917,702	South Korea	National Health Insurance Service (NHIS)	2009–2015	56.8 (6.9)	788175 (41.1)	5.1 y	Diabetes-only cohort: adults aged ≥40 years with diabetes	DR	Age, sex, smoking, alcohol, exercise, income, glucose, diabetes duration, BMI, dyslipidemia, hypertension, CKD, stroke, IHD, depression, meds	Diabetic retinopathy was significantly associated with the development of all-cause dementia (HR 1.09), Alzheimer’s Disease (HR 1.10), and Vascular Dementia (HR 1.08)
Hwang 2021 (32)	Prospective cohort study	3,375	United States	Cardiovascular Health Study (CHS) and Medicare Part B claims	Baseline 1989-93; Cognition Study 1998-99	74.8 (9.5)	NA	4.1 y	Population-based cohort with explicitly identified DR exposure and a study-defined reference group; not an exclusively diabetes-only DR-versus-no-DR comparison	DR	Demographics, cardiovascular risk factors, APOE genotype, and other ophthalmic conditions	Diabetic retinopathy was significantly associated with an increased risk of Vascular Dementia/mixed dementia (HR 2.63), but not Alzheimer’s Disease or all-cause dementia in this cohort
Shang 2023 (35)	Prospective cohort study	112364	United Kingdom	UK Biobank	Baseline 2006–2010; Follow-up to 2021	62.4 (7.3)	60676 (54)	11 y	Population-based cohort; DR-specific analysis restricted to participants with diabetes used	DR	Age, gender, education, income, smoking, alcohol, physical activity, BMI, cholesterol, glucose, diet	Diabetes-related eye disease (DRED) was independently associated with an increased risk of incident dementia (HR 1.61, 95% CI 1.30–2.00) among patients with diabetes

DR, diabetic retinopathy; DMR, diabetic retinopathy/diabetic retinal disease; PDR, proliferative diabetic retinopathy; MCI, mild cognitive impairment; OR, odds ratio; HR, hazard ratio; CI, confidence interval; NR, not reported. The reported overall cohort size may differ from the analytical denominator contributing to a particular outcome. The crude OR synthesis used event counts extracted from the original reports, whereas the longitudinal synthesis used study-reported adjusted HRs. Population-based studies were retained only when an explicitly DR-related or diabetes-restricted estimate relevant to the review question was available. Random-effects weights are those shown in the corresponding forest plots.

### 3.3 Risk of bias

Using the NIH Quality Assessment Tool for Observational Cohort and Cross-Sectional Studies, 12 studies were rated as good quality and two as fair quality; no study was rated poor (**Supplementary Table S1**; **Supplementary Figure S1**).

### 3.4 Results of synthesis

#### 3.4.1 Quantitative syntheses of diabetic retinopathy and cognitive outcomes

Seven studies, Bruce, Crosby-Nwaobi, Gao, Hendrie, Kimura, Murata, and Xia, contributed crude dichotomous event data. The pooled analysis did not demonstrate a statistically significant association between DR and the study-defined cognitive impairment or dementia outcomes (OR 1.07, 95% CI 0.94–1.23; p=0.310; I^2^=52.3%; τ^2^=0.0005; 95% prediction interval 0.90–1.28; **Figure 2**; **Table 1**). Seven studies, Deal, Exalto, Lee, Rodill, Yu, Hwang, and Shang, contributed adjusted longitudinal hazard estimates for incident dementia. Each study contributed an incident dementia endpoint as defined in its report; the study-specific endpoint and ascertainment are shown in **Tables 2** and **1**. DR was associated with a higher incidence of dementia (HR 1.55, 95% CI 1.19–2.02; p=0.001; I^2^=97.8%; τ^2^=0.1073; 95% prediction interval 0.65–3.68; **Figure 3**; **Table 1**). The prediction interval crossed the null value, indicating that the magnitude and statistical significance of the association may differ substantially across future settings. Leave-one-out pooled estimates ranged from HR 1.36 (95% CI 1.13–1.63) after omitting Lee et al. to HR 1.66 (95% CI 1.26–2.20) after omitting Yu et al.; no single-study omission changed the direction or statistical significance of the association (**Supplementary Table S3**).

**Figure 2:**
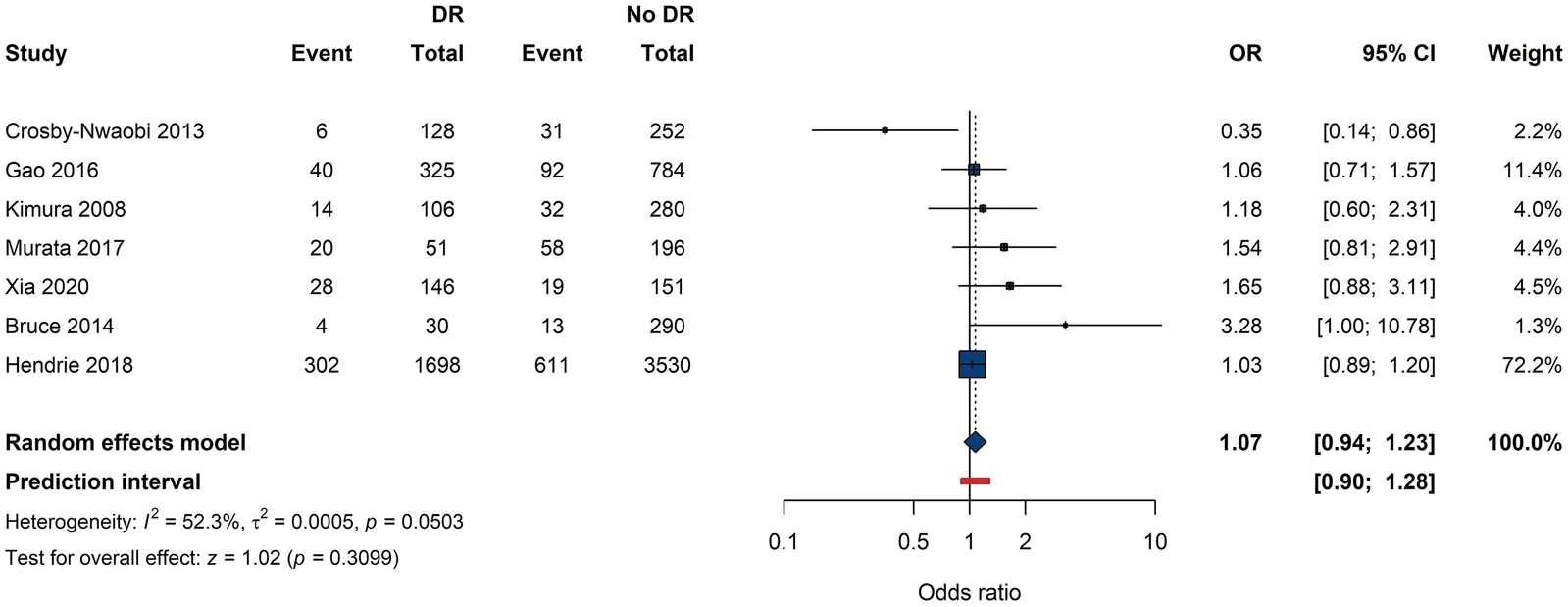
Forest plot of the crude dichotomous association between diabetic retinopathy and cognitive impairment or dementia. This forest plot displays the odds ratios (OR) for study-defined cognitive impairment or dementia in participants with versus without diabetic retinopathy. The analysis pools data across seven observational studies.

**Figure 3:**
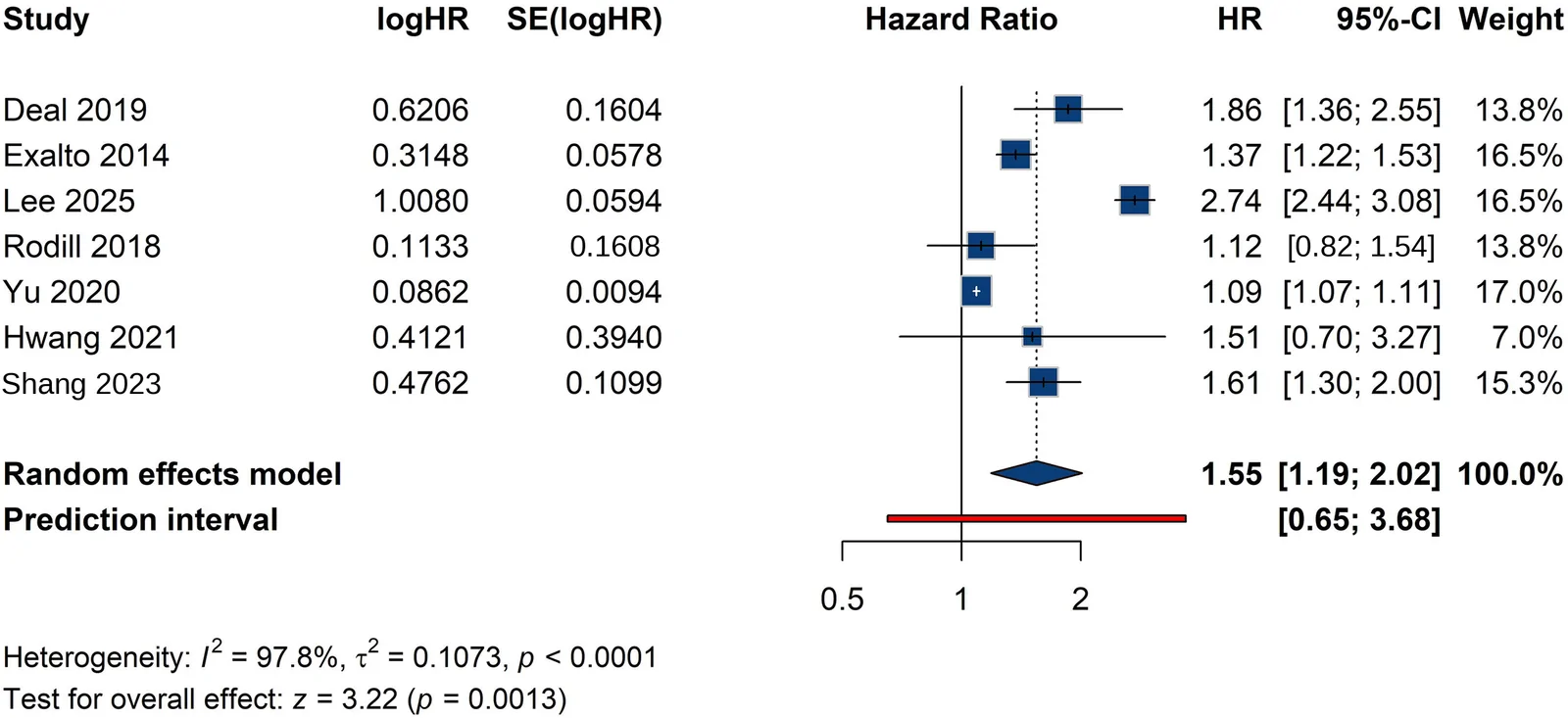
Forest plot of adjusted longitudinal hazard ratios for incident dementia associated with diabetic retinopathy. This forest plot presents the pooled adjusted hazard ratios (HR) for incident dementia across seven longitudinal cohort studies.

#### 3.4.2 Association between diabetic retinopathy and dementia subtypes

Evidence concerning dementia subtypes was inconsistent. Hwang et al. reported that DR was associated with vascular or mixed dementia (HR 2.63, p=0.029) but not Alzheimer’s disease (HR 0.46, p=0.439); however, these estimates were based on relatively few DR-exposed participants and events (32). Deal et al. similarly reported an association between moderate/severe DR and cerebrovascular-related dementia or MCI (HR 2.29, p=0.008), whereas the estimate for Alzheimer’s-related dementia or MCI was not statistically significant (HR 1.33, p=0.452) (26). This pattern is compatible with a small-vessel-disease hypothesis but does not establish an underlying mechanism.

Conversely, Lee et al. reported associations with both Alzheimer’s disease (HR 2.89, 95% CI 2.50–3.34) and vascular dementia (HR 2.37, 95% CI 1.40–4.02) (37). Yu et al. also reported associations with Alzheimer’s disease (HR 1.10, 95% CI 1.07–1.12) and vascular dementia (HR 1.08, 95% CI 1.03–1.14) (36). Because definitions, populations, and analytical models differed across studies, these findings were not pooled and do not establish a consistent subtype-specific association.

## 4 Discussion

The present meta-analysis found no statistically significant association in the crude dichotomous synthesis, whereas adjusted longitudinal evidence indicated an association between diabetic retinopathy and a higher incidence of dementia (HR 1.55). The longitudinal synthesis included incident dementia outcomes rather than general cognitive impairment. The Hwang and Lee estimates arose from population-based cohorts and should not be interpreted as direct diabetes-only DR-versus-no-DR comparisons. This temporal distinction suggests that DR may be a prognostic marker for future cognitive deterioration rather than merely coexisting with established impairment, potentially reflecting shared pathophysiology that precedes clinically detectable cognitive deficits. In a systematic review, Dove et al. found that, after a mean follow-up duration of 9.2 years, 29.6% of diabetic patients who were cognitively intact at baseline developed CI without dementia (CIND), which is a category for those who show signs of cognitive decline without fitting the criteria for dementia. In the CIND-at-baseline cohort, 22.1% were diagnosed with dementia after a mean follow-up duration of 7.7 years. They noted that progression to dementia was associated with worse glycemic control (HbA1c >7.5%; HR 2.87, 95% CI: 1.20; 6.85) (4).

In a prior systematic review, Chan et al. identified a statistically significant association between DR and any type of cognitive decline from five studies with longitudinal data (Relative risk [RR] 1.31). From cross-sectional data, 18 studies found that the odds of any CI (OR 1.48) and early-stage CI (OR 1.59) were significantly higher among patients with DR compared to those without DR; however, significant heterogeneity limited the reliability of these estimates (19). Similarly, Cheng et al. pooled data from ten studies and detected a statistically meaningful increase in the odds of CI in patients with DR (OR 2.24, 95% CI: 1.89; 2.66). Subgrouping of studies based on adjustment for confounders revealed similar odds of CI with DR (OR 2.12, 95% CI: 1.55; 2.88). In two studies, where patients were followed up for more than ten years, the odds were significantly higher (OR 2.92) with no detectable heterogeneity. Nevertheless, these estimates were pooled from a small number of studies encompassing a relatively small number of patients, so cautious interpretation is warranted (18). Based on data from four studies, Feng et al. reported a pooled relative risk of AD of 1.21 among patients with DR (95% CI: 1.04; 1.41). Conversely, the association between DR and the risk of VaD did not reach statistical significance in the pooled analysis (RR 1.44, 95% CI: 0.93; 2.23) (20). Differences between those estimates and the present findings may reflect our exclusion of nonspecific retinopathy, verification of DR-specific or diabetes-restricted estimates in population-based cohorts, separation of crude dichotomous ORs from adjusted longitudinal HRs, and inclusion of the 2025 Lee cohort. ORs, RRs, and HRs are not mathematically equivalent and were therefore compared only contextually; in particular, an OR may overstate an RR when the outcome is common.

The observed association may reflect shared retinal and cerebral microvascular pathology. Previous studies have associated DR with cerebral small-vessel-disease markers, structural brain changes, and subsequent cognitive decline (39–42). Hyperglycemia-related endothelial dysfunction, oxidative stress, inflammation, vascular endothelial growth factor, and advanced glycation end products have been proposed as processes affecting both retinal and cerebral neurovascular tissues (43, 44). Neurodegenerative pathways involving amyloid and tau have also been proposed (10, 11, 45–48); however, these biomarkers were not evaluated in the present meta-analysis, and these mechanisms should be regarded as hypotheses rather than findings of this review. The available evidence does not establish causality or demonstrate that routine cognitive screening based solely on DR status improves clinical outcomes.

Our findings regarding dementia subtypes clarified a relationship between microvascular and neurodegenerative processes. Several large population-based studies identified strong associations between DR and VaD or cerebrovascular-related CI. These observations align with the retinal/cerebral small vessel disease hypothesis, which posits that DR represents a visible marker of systemic microangiopathy affecting end-organs, including the brain (41). However, conflicting evidence from nationwide Korean cohort studies challenges this dichotomous perspective. Lee et al. and Yu et al. reported significant associations between DR and both AD and VaD, with comparable effect sizes across subtypes (36, 37).

Gupta et al. hypothesized that the shared embryological origin of cerebral and retinal blood barriers may make them both vulnerable to similar deterioration in diabetes (42). Another hypothesis reported in the work of Arden et al. indicates that hyperglycemia in diabetes leads to endothelial dysfunction, causing hypoxia and ischemia by means of vascular endothelial growth factor (VEGF), advanced glycation end products (AGEs), oxidative stress, and inflammatory processes, which occur both in the cerebral and retinal vessels (43). Gorska-Ciebiada et al. inferred a notable elevation in serum AGEs in diabetic patients with MCI compared to controls. Receptors for AGEs (RAGE) bind AGEs and inflate the extent of oxidative stress and inflammation by activation of nuclear factor kappa B (NF-kB) in neurovascular tissues in the retina and the brain (44).

There are various hypotheses regarding the interaction between diabetes and AD. For instance, the hyperinsulinemic state in early type 2 DM has been implicated in the deposition of amyloid-beta in neural tissues due to the consumption of insulin-degrading enzyme (IDE) by high insulin levels. IDE is responsible for the degradation and clearance of amyloid-beta, the accumulation of which leads to the formation of senile plaques in the brain, causing AD (45–47). Moreover, Zhu et al. posited another mechanism related to the other histopathological aspect of AD, the neurofibrillary tangles (NFTs), which consist of altered microtubule-related tau protein (48). Their hypothesis indicates that glycogen synthase kinase 3β (GSK3β)-mediated hyperphosphorylation of tau protein, in patients with DR, contributes to synaptic degeneration in retinal ganglion cells (RGCs) prior to microvascular changes, similar to cerebral changes in AD (11). This shared neurotoxic mechanism seems plausible considering that retinal neurodegenerative changes occur much earlier than microvascular changes in the diabetic eye (10).

From a research perspective, the heterogeneity in subtype-specific associations emphasized the need for standardized neuropathological confirmation in epidemiologic studies. Future investigations should employ multimodal biomarker approaches, including amyloid and tau imaging, vascular MRI markers, and retinal imaging with quantitative microvascular assessment, to disentangle the relative contributions of vascular and neurodegenerative pathology. Several limitations should be considered. First, all included studies were observational; residual and unmeasured confounding cannot be excluded, and the covariates included in adjusted models varied substantially. Second, considerable clinical and methodological heterogeneity existed in population composition, DR definition and severity, dementia definition and ascertainment, follow-up duration, and adjustment strategy. In particular, Hwang et al. and Lee et al. were population-based cohorts using study-defined reference groups, which limits direct comparability with diabetes-only cohorts. Only one included study specifically evaluated type 1 diabetes, and the evidence therefore predominantly reflects type 2 diabetes. The inconsistent severity categories prevented a reliable dose–response synthesis; although Lee et al. reported associations for both moderate and severe DMR, the available studies did not demonstrate a consistent severity gradient. Third, only seven studies contributed to each quantitative synthesis. Although the leave-one-out analysis did not identify a single influential study, reliable subgroup analysis and meta-regression were not performed, and the extreme longitudinal heterogeneity and prediction interval crossing the null limit confidence in the pooled estimate. Fourth, formal funnel-plot asymmetry testing was underpowered, and publication bias could not be adequately assessed. A formal GRADE certainty assessment was not undertaken, which should also be considered when interpreting the strength of the evidence. Fifth, administrative coding and medical-record ascertainment in several studies may have introduced both exposure and outcome misclassification. Finally, restriction to English-language full-text publications and the absence of a systematic grey-literature search may have introduced language and publication biases.

## 5 Conclusion

In conclusion, the crude dichotomous synthesis did not demonstrate a statistically significant association between DR and cognitive impairment or dementia, whereas adjusted longitudinal evidence suggested that DR was associated with a higher incidence of dementia. However, the observational study designs, residual confounding, extreme longitudinal heterogeneity, a prediction interval crossing the null, and possible exposure and outcome misclassification limit confidence and require cautious interpretation. Evidence concerning Alzheimer’s disease and vascular dementia remains inconsistent. These findings do not establish causality or the benefit of routine cognitive screening based solely on DR status.

## Data Availability

The original contributions presented in the study are included in the article/**Supplementary Material**. Further inquiries can be directed to the corresponding author.

## Supplementary material

Supplementary Figure 1 NIH quality assessment of the included observational cohort studies. Methodological quality was evaluated using the National Institutes of Health (NIH) Quality Assessment Tool for Observational Cohort and Cross-Sectional Studies. Green circles with a plus sign (+) indicate that the criterion was met (“Yes”), red circles with a minus sign (−) indicate that the criterion was not met (“No”), and yellow circles with a question mark ()? indicate that the information was not reported or could not be determined. The final column shows the overall study-quality assessment (H, high quality; M, moderate quality).
