# Supplementary material for: The association between diabetic retinopathy and cognitive impairment in patients with diabetes mellitus: a systematic review and meta-analysis
Source: Front Endocrinol (Lausanne). 2026 Oct 15;17:1881602. doi: 10.3389/fendo.2026.1881602 (PMC13716637; doi:10.3389/fendo.2026.1881602)
Supplement: Supplementary file 1 [file Table1.docx]

Supplementary Table S1 Database Specific Search Strategies

**Last search date for all formal databases: 10 December 2025**

The formal systematic search covered PubMed, Scopus, Web of Science Core Collection, and the Cochrane Library from inception through 10 December 2025. The strategy used two concept blocks: diabetic retinopathy and cognitive impairment or dementia.

# PubMed

**Platform: National Center for Biotechnology Information | Last searched: 10 December 2025**

| **Line** | **Search strategy** |
| --- | --- |
| #1 | "Diabetic Retinopathy"[Mesh] |
| #2 | ("diabetic retinopath*"[Title/Abstract] OR "diabetic retinal disease*"[Title/Abstract] OR "diabetic eye disease*"[Title/Abstract] OR "diabetic macular edem*"[Title/Abstract] OR "diabetic macular oedem*"[Title/Abstract] OR NPDR[Title/Abstract] OR PDR[Title/Abstract]) |
| #3 | ((diabet*[Title/Abstract] OR T1DM[Title/Abstract] OR T2DM[Title/Abstract]) AND (retinopath*[Title/Abstract] OR "retinal microvascular"[Title/Abstract] OR "retinal vascular"[Title/Abstract])) |
| #4 | #1 OR #2 OR #3 |
| #5 | ("Cognitive Dysfunction"[Mesh] OR "Mild Cognitive Impairment"[Mesh] OR "Neurocognitive Disorders"[Mesh] OR "Dementia"[Mesh] OR "Alzheimer Disease"[Mesh] OR "Dementia, Vascular"[Mesh]) |
| #6 | (cognit*[Title/Abstract] OR dement*[Title/Abstract] OR alzheimer*[Title/Abstract] OR neurocognit*[Title/Abstract] OR "memory impair*"[Title/Abstract] OR "memory declin*"[Title/Abstract] OR "memory loss"[Title/Abstract] OR MCI[Title/Abstract] OR CIND[Title/Abstract] OR MMSE[Title/Abstract] OR MoCA[Title/Abstract]) |
| #7 | #5 OR #6 |
| #8 | #4 AND #7 |

**Limits:** Database inception to 10 December 2025.

# Scopus

**Platform: Elsevier | Last searched: 10 December 2025**

| **Line** | **Search strategy** |
| --- | --- |
| #1 | TITLE-ABS-KEY("diabetic retinopath*" OR "diabetic retinal disease*" OR "diabetic eye disease*" OR "diabetic macular edem*" OR "diabetic macular oedem*" OR NPDR OR PDR OR ((diabet* OR T1DM OR T2DM) W/3 (retinopath* OR "retinal microvascular" OR "retinal vascular"))) |
| #2 | TITLE-ABS-KEY(cognit* OR dement* OR alzheimer* OR neurocognit* OR "memory impair*" OR "memory declin*" OR "memory loss" OR MCI OR CIND OR MMSE OR MoCA) |
| #3 | #1 AND #2 |

**Limits:** Database inception to 10 December 2025.

# Web of Science Core Collection

**Platform: Clarivate | Last searched: 10 December 2025**

| **Line** | **Search strategy** |
| --- | --- |
| #1 | TS=("diabetic retinopath*" OR "diabetic retinal disease*" OR "diabetic eye disease*" OR "diabetic macular edem*" OR "diabetic macular oedem*" OR NPDR OR PDR OR ((diabet* OR T1DM OR T2DM) NEAR/3 (retinopath* OR "retinal microvascular" OR "retinal vascular"))) |
| #2 | TS=(cognit* OR dement* OR alzheimer* OR neurocognit* OR "memory impair*" OR "memory declin*" OR "memory loss" OR MCI OR CIND OR MMSE OR MoCA) |
| #3 | #1 AND #2 |

**Limits:** Database inception to 10 December 2025.

# Cochrane Library

**Platform: Wiley Cochrane Library Search Manager | Last searched: 10 December 2025**

| **Line** | **Search strategy** |
| --- | --- |
| #1 | MeSH descriptor: [Diabetic Retinopathy] explode all trees |
| #2 | ("diabetic retinopath*" OR "diabetic retinal disease*" OR "diabetic eye disease*" OR "diabetic macular edem*" OR "diabetic macular oedem*" OR NPDR OR PDR):ti,ab,kw |
| #3 | ((diabet* OR T1DM OR T2DM) NEAR/3 (retinopath* OR "retinal microvascular" OR "retinal vascular")):ti,ab,kw |
| #4 | #1 OR #2 OR #3 |
| #5 | MeSH descriptor: [Cognitive Dysfunction] explode all trees |
| #6 | MeSH descriptor: [Mild Cognitive Impairment] explode all trees |
| #7 | MeSH descriptor: [Neurocognitive Disorders] explode all trees |
| #8 | MeSH descriptor: [Dementia] explode all trees |
| #9 | MeSH descriptor: [Alzheimer Disease] explode all trees |
| #10 | MeSH descriptor: [Dementia, Vascular] explode all trees |
| #11 | (cognit* OR dement* OR alzheimer* OR neurocognit* OR "memory impair*" OR "memory declin*" OR "memory loss" OR MCI OR CIND OR MMSE OR MoCA):ti,ab,kw |
| #12 | #5 OR #6 OR #7 OR #8 OR #9 OR #10 OR #11 |
| #13 | #4 AND #12 |

**Limits:** Database inception to 10 December 2025. No language, publication-type, or study-design filter applied.

# Abbreviations and reporting notes

- CIND, cognitive impairment no dementia; MCI, mild cognitive impairment; MMSE, Mini-Mental State Examination; MoCA, Montreal Cognitive Assessment; NPDR, nonproliferative diabetic retinopathy; PDR, proliferative diabetic retinopathy; T1DM, type 1 diabetes mellitus; T2DM, type 2 diabetes mellitus.
- English-language eligibility was applied during screening rather than as a database search filter.
- A preliminary Embase scoping search was used only to identify terminology. Embase was not part of the formal retrieval set and was not included in the PRISMA record counts.
- Conference abstracts, reviews, and other ineligible publication types were removed during screening rather than by database filters.
