# Supplementary material for: The association between diabetic retinopathy and cognitive impairment in patients with diabetes mellitus: a systematic review and meta-analysis
Source: Front Endocrinol (Lausanne). 2026 Oct 15;17:1881602. doi: 10.3389/fendo.2026.1881602 (PMC13716637; doi:10.3389/fendo.2026.1881602)
Supplement: Supplementary file 2 [file Table2.docx]

**Supplementary Table 2 Risk Of Bias**

| Study | 1. Was the research question or objective in this paper clearly stated? | 2. Was the study population clearly specified and defined? | 3. Was the participation rate of eligible persons at least 50%? | 4. Were all the subjects selected or recruited from the same or similar populations (including the same time period)? Were inclusion and exclusion criteria for being in the study prespecified and applied uniformly to all participants? | 5. Was a sample size justification, power description, or variance and effect estimates provided? | 6. For the analyses in this paper, were the exposure(s) of interest measured prior to the outcome(s) being measured? | 7. Was the timeframe sufficient so that one could reasonably expect to see an association between exposure and outcome if it existed? | 8. For exposures that can vary in amount or level, did the study examine different levels of the exposure as related to the outcome (e.g., categories of exposure, or exposure measured as continuous variable)? | 9. Were the exposure measures (independent variables) clearly defined, valid, reliable, and implemented consistently across all study participants? | 10. Was the exposure(s) assessed more than once over time? | 11. Were the outcome measures (dependent variables) clearly defined, valid, reliable, and implemented consistently across all study participants? | 12. Were the outcome assessors blinded to the exposure status of participants? | 13. Was loss to follow-up after baseline 20% or less? | 14. Were key potential confounding variables measured and adjusted statistically for their impact on the relationship between exposure(s) and outcome(s)? | Overall Rating |
| --- | --- | --- | --- | --- | --- | --- | --- | --- | --- | --- | --- | --- | --- | --- | --- |
| Bruce 2014 | Yes | Yes | NR | Yes | Yes | Yes | Yes | Yes | Yes | No | Yes | NR | Yes | Yes | High Quality |
| Crosby-Nwaobi 2013 | Yes | Yes | NR | Yes | No | Yes | Yes | Yes | Yes | No | Yes | NR | Yes | Yes | High Quality |
| Deal 2019 | Yes | Yes | Yes | Yes | No | Yes | Yes | Yes | Yes | No | Yes | NR | Yes | Yes | High Quality |
| Exalto 2014 | Yes | Yes | NR | Yes | No | Yes | Yes | Yes | Yes | No | Yes | NR | Yes | Yes | High Quality |
| Gao 2016 | Yes | Yes | NR | Yes | No | Yes | Yes | Yes | Yes | No | Yes | NR | Yes | Yes | High Quality |
| Hendrie 2018 | Yes | Yes | Yes | Yes | Yes | Yes | Yes | Yes | Yes | No | Yes | NR | Yes | Yes | High Quality |
| Kimura 2008 | Yes | Yes | NR | Yes | No | No | No | Yes | Yes | No | Yes | NR | Yes | Yes | Moderate Quality |
| Lee 2025 | Yes | Yes | Yes | Yes | Yes | Yes | Yes | Yes | Yes | No | Yes | NR | Yes | Yes | High Quality |
| Murata 2017 | Yes | Yes | NR | Yes | No | Yes | Yes | Yes | Yes | No | Yes | NR | Yes | Yes | High Quality |
| Rodill 2018 | Yes | Yes | Yes | Yes | No | Yes | Yes | Yes | Yes | No | Yes | NR | Yes | Yes | High Quality |
| Xia 2020 | Yes | Yes | No | Yes | No | No | No | Yes | Yes | No | Yes | NR | Yes | Yes | Moderate Quality |
| Yu 2020 | Yes | Yes | Yes | Yes | Yes | Yes | Yes | Yes | Yes | No | Yes | NR | Yes | Yes | High Quality |
| Hwang 2021 | Yes | Yes | Yes | Yes | Yes | Yes | Yes | Yes | Yes | No | Yes | NR | Yes | Yes | High Quality |
| Shang 2021 | Yes | Yes | Yes | Yes | Yes | Yes | Yes | Yes | Yes | No | Yes | NR | Yes | Yes | High Quality |
