# Supplementary material for: The association between diabetic retinopathy and cognitive impairment in patients with diabetes mellitus: a systematic review and meta-analysis
Source: Front Endocrinol (Lausanne). 2026 Oct 15;17:1881602. doi: 10.3389/fendo.2026.1881602 (PMC13716637; doi:10.3389/fendo.2026.1881602)
Supplement: Supplementary file 3 [file Table3.docx]

**Supplementary Table S3 Leave One Out Sensitivity Analysis**

The longitudinal random-effects model was refitted after omitting each study in turn. All estimates remained above the null, indicating that no single study accounted for the direction or statistical significance of the pooled association.

| Study omitted | Pooled HR | 95% CI | τ² |
| --- | --- | --- | --- |
| Deal 2019 | 1.50 | 1.11–2.03 | 0.1219 |
| Exalto 2014 | 1.58 | 1.16–2.16 | 0.1274 |
| Lee 2025 | 1.36 | 1.13–1.63 | 0.0339 |
| Rodill 2018 | 1.63 | 1.22–2.17 | 0.1093 |
| Yu 2020 | 1.66 | 1.26–2.20 | 0.0953 |
| Hwang 2021 | 1.55 | 1.16–2.06 | 0.1180 |
| Shang 2021 | 1.54 | 1.12–2.10 | 0.1297 |

Abbreviations: CI, confidence interval; HR, hazard ratio. Models used inverse-variance random effects with restricted maximum-likelihood estimation of τ², consistent with the primary longitudinal analysis.
